# Peer health education for injury prevention: a cost-effective measure that can spread medical knowledge amongst children and youths

**DOI:** 10.1186/1757-7241-21-S1-S23

**Published:** 2013-05-28

**Authors:** GC Strack Neves, A Nasr, P Abreu-Reis, JG Trindade de Abreu, PA Trindade Abreu, B Scheffer, F Tomasich, I Collaço

**Affiliations:** 1 Médico pela UFPR - Turma 134. Brazil

## Introduction and objectives

Domestic accidents yearly account for an important amount of morbidity related to trauma worldwide, mainly amongst children and youth. One of the best ways to address this issue is through incorporating preventive measures to the daily routine of schools. It is the aim of this study to introduce a cost-effective peer-education preventive action.

## Methods

A prospective interventional non-randomized comparative multicentric study of students from two basic schools in Brazil. A questionnaire with 30 true or false questions was applied by volunteer medical students in two cities from May to June 2012 before and after a peer-educational lecture on prevention of domestic injuries. Data collected was divided into two groups: those from São João del-Rei (SJ group), and those from Ponta Grossa (PG group). Statistical analysis was performed using the chi-square for discrete, and the students’ t-test for continuous variables.

## Results

Of 732 students who answered the questionnaire, 239 were from SJ group and 493 from PG group. The mean age was 13.82 y-o. 55.87% of students reported previous domestic injuries that required medical care. More than 95% had the primary care performed by a person rather than a paramedic. About 1 of each 3 student did not know past immunization, and 51% knew nothing about tetanus vaccine. There was no difference in their pre-test assessment (20.85 for SJ vs 21 for PG, p=0.6007). There was a higher number of correct answers in the post-test than in the pre-test (25.28vs20.49, p<0.0000001). Both SJ and PG groups did better after the peer lecture in terms of correct answers (20.85vs24.87, p<0.0000001; 21vs25.93, p<0.0000001, respectively). However, there was an important difference in the post-tests (24.87vs25.93, p=0.000000913). The estimated overall cost was 50 dollars.

## Conclusion

Peer-educational strategies are cost-effective for spreading medical knowledge amongst children and youth. It is absolutely feasible to reproduce this model in different scenarios.

